# Interdisziplinäre multimodale Schmerztherapie: Macht die Dosis einen Unterschied? Ein Vergleich aus der klinischen Routineversorgung

**DOI:** 10.1007/s00482-024-00838-6

**Published:** 2024-10-09

**Authors:** Philipp Baumbach, Peter Storch, Thomas Weiss, Winfried Meissner, Fabian Rottstädt

**Affiliations:** 1 Universitätsklinikum Jena, Klinik für Anästhesiologie und Intensivmedizin, Friedrich-Schiller-Universität Jena, Am Klinikum 1, 07747 Jena, Deutschland; 2 Universitätsklinikum Jena, Klinik für Neurologie, Friedrich-Schiller-Universität Jena, Jena, Deutschland; 3 Lehrstuhl für Klinische Psychologie, Friedrich-Schiller-Universität, Am Steiger 3, 07743 Jena, Deutschland; 4 Abteilung Palliativmedizin der Klinik für Innere Medizin II, Universitätsklinikum Jena, Jena, Deutschland

**Keywords:** Interdisziplinäre multimodale Schmerztherapie, Chronischer Schmerz, Schmerz, Schmerztherapie, Behandlungssetting, Therapiedauer, Dosierung, Interdisciplinary multimodal pain therapy, Chronic pain, Pain, Pain management, Treatment setting, Duration of therapy, Dosage

## Abstract

**Hintergrund:**

Die interdisziplinäre multimodale Schmerztherapie (IMST) gilt als etablierte Behandlung bei Patient:innen mit starken chronischen Schmerzen. Über die Rolle der Dosierung der Behandlung und insbesondere hinsichtlich des Zusammenhangs der Dauer der IMST und des Behandlungserfolgs liegen kaum Erkenntnisse vor.

**Ziel:**

Ziel dieser retrospektiven Studie war es, den mittelfristigen Behandlungserfolg einer kurzen stationären (*KST*, 1 Woche) und einer langen tagesklinischen (*LTT*, 4 Wochen) IMST mit vergleichbarem inhaltlichem Behandlungskonzept und vergleichbarer Therapieintensität (20 h/Woche) bei Patient:innen mit starken chronischen Schmerzen zu vergleichen.

**Methoden:**

Patient:innen beider Gruppen beantworteten jeweils zu Beginn und zum Ende der IMST sowie nach 3 Monaten den Deutschen Schmerzfragebogen. Die primären Zielgrößen umfassten die schmerzbedingte Beeinträchtigung und die durchschnittliche Schmerzintensität im Follow-up bei zu Therapiebeginn hinsichtlich Geschlecht, Alter, Schmerzintensität und -beeinträchtigung vergleichbaren Patient:innen.

**Ergebnisse:**

Während initial beide Gruppen signifikante Behandlungseffekte in der schmerzbedingten Beeinträchtigung und durchschnittlichen Schmerzintensität zeigten, berichteten im 3‑Monats-Follow-up Patient:innen der *LTT* (*n* = 32) signifikant bessere Werte in beiden Variablen im Vergleich zu Patient:innen der *KST* (*n* = 32). Dies war auf anhaltende positive Effekte bei *LTT*-Patient:innen und eine Verschlechterung in der *KST*-Gruppe zurückzuführen.

**Schlussfolgerung:**

Die Ergebnisse deuten darauf hin, dass initiale Behandlungseffekte in beiden Behandlungssettings zu beobachten sind, eine längere Therapiedauer aber scheinbar die langfristige Stabilität der Behandlungseffekte begünstigt.

**Graphic abstract:**

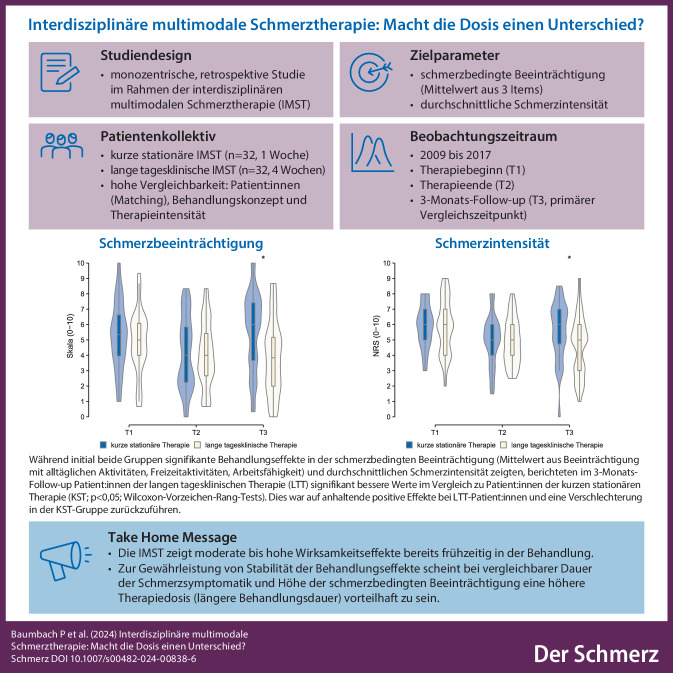

**Zusatzmaterial online:**

Zusätzliche Informationen sind in der Online-Version dieses Artikels (10.1007/s00482-024-00838-6) enthalten.

## Abstract (en)

**Background:**

Interdisciplinary multimodal pain therapy (IMPT) is an established treatment for patients with severe chronic pain. Little evidence is available on the role of treatment dosage and, in particular, on the association between the duration of IMPT and treatment outcome.

**Aim:**

The aim of this retrospective study was to compare the medium-term treatment success of a short inpatient (*SIT*, 1 week) and a long outpatient (*LOT*, 4 weeks) IMPT with a comparable treatment concept and comparable therapy intensity (20 h/week) in patients with severe chronic pain.

**Methods:**

Patients in both groups completed the German Pain Questionnaire at the beginning and end of IMPT as well as after 3 months. Primary outcome measures included pain-related impairment and average pain intensity at follow-up in patients of comparable sex, age as well as pain intensity and impairment at the beginning of the therapy.

**Results:**

While both groups initially showed significant treatment effects in pain-related impairment and average pain intensity, *LOT* patients (*n* = 32) reported significantly better values in both variables at 3‑month follow-up compared with *SIT* patients (*n* = 32). This was due to sustained positive effects in *LOT* patients and worsening in the *SIT* group.

**Conclusion:**

The results indicate that initial treatment effects can be observed in both treatment settings, but a longer duration of therapy seems to favour the long-term stability of treatment effects.

## Hintergrund und Fragestellung

Chronische Schmerzen sind weit verbreitet und mit erheblichen individuellen, sozialen und gesellschaftlichen Auswirkungen verbunden [3, 15]. In einer Vielzahl von Studien und Metaanalysen zeigte sich die interdisziplinäre multimodale Schmerztherapie (IMST) als effiziente und wirksame Behandlungsmethode für chronische Schmerzen [1, 4, 11, 12].

Mehrere Übersichtsarbeiten haben den Zusammenhang zwischen Dosierungsaspekten und der Wirksamkeit von IMST-Programmen für Patient:innen mit chronischen Rückenschmerzen diskutiert. Der Begriff „Dosierung“ bezieht sich in diesen Arbeiten hauptsächlich auf die Therapiedauer (Gesamtzahl der Kontaktstunden) und die Therapieintensität (Anzahl der wöchentlichen Kontaktstunden). Eine Übersichtsarbeit kam zu dem Schluss, dass Programme mit täglichem Kontakt und einer Behandlungsdauer von mehr als 100 h im Vergleich zu Programmen mit 1–2 Kontakten pro Woche und weniger als 30 h Behandlung oder unimodalen Behandlungen eine höhere Wirksamkeit erzielen [2, 7]. Zudem konnte in einer anderen Übersichtsarbeit die überlegene langfristige Wirksamkeit von intensiveren, stationären im Vergleich zu weniger intensiven, ambulanten Behandlungen herausgearbeitet werden [22]. Eine weitere systematische Übersichtsarbeit konnte ebenfalls einen positiven Zusammenhang zwischen der Therapiedauer (Anzahl der Kontaktstunden) und dem Therapieergebnis darstellen. Jedoch konnte kein unabhängiger Effekt der Therapiedauer nachgewiesen werden, da diese mit inhaltlichen Aspekten der Therapie, wie etwa der Anzahl der beteiligten Disziplinen bzw. Therapieinhalte konfundiert war [26]. In einer ähnlichen Übersichtsarbeit konnten ebenfalls keine allgemeinen Effekte der Dosierung, in diesem Fall der Gesamttherapiedauer (< 5 Wochen *vs.* ≥ 5 Wochen), täglicher *vs.* nichttäglicher Kontakte sowie hoher *vs.* niedriger Intensität (< 30 *vs.* ≥ 30 Kontaktstunden pro Woche), identifiziert werden [5].

Direkte Vergleiche verschiedener Dosierungen der IMST sind besonders vor dem Hintergrund von Wirksamkeitsforschung bzw. Kosten-Wirksamkeits-Aspekten der IMST interessant [26]. Das primäre Ziel dieser retrospektiven Studie am Universitätsklinikum Jena war es zu analysieren, ob bei vergleichbaren Behandlungsmethoden/-inhalten und Patientenpopulationen eine lange tagesklinische Behandlung im Vergleich zu einer kurzen stationären Behandlung mit spezifischen mittelfristigen Therapieergebnissen assoziiert ist.

## Methodik

### IMST-Dosierung und Behandlungssetting

Die Zuweisung der Patient:innen erfolgte durch Haus- oder Fachärzt:innen oder durch Ambulanzen bzw. Polikliniken des Krankenhauses. Zur Klärung der Therapieindikation und -motivation fand vor der Behandlung ein interdisziplinäres Screening statt. Dieses umfasst ein schmerztherapeutisches Aufnahmegespräch, physiotherapeutisch-ärztliche Untersuchungen, schmerzpsychologische und pflegerische Anamnese sowie Fragebogendiagnostik. Die Durchführung der IMST erfolgte am Universitätsklinikum Jena. Das Follow-up fand 3 Monate nach Therapieende statt.

In der ersten Gruppe durchliefen die Patient:innen eine stationäre IMST mit insgesamt 7 Behandlungstagen (1 Woche, kurze stationäre Therapie [KST]). Diese erfolgte einzeln oder in Gruppen mit bis zu 4 Patient:innen zwischen Januar 2009 und November 2010. Insgesamt belief sich die Dauer der therapeutischen Interventionen auf etwa 20 h (Therapiedosis, vgl. *Online-Zusatzmaterial: Tabelle A1*) und die Dauer der interdisziplinären Teamsitzungen auf 2–3 h pro Therapiegruppe.

Patient:innen der zweiten Gruppe wurden an 20 Tagen über einen Zeitraum von 4 Wochen in der Tagesklinik (lange tagesklinische Therapie [LTT]) behandelt. Die LTT wurde in Gruppen von 5 bis 9 Patient:innen zwischen Juni 2013 und März 2017 durchgeführt, wobei sich die Dauer der therapeutischen Interventionen auf insgesamt etwa 80 h belief (Therapiedosis, vgl. *Online-Zusatzmaterial: Tabelle A1*). Die Dauer der interdisziplinären Teamsitzungen belief sich auf 14–15 h pro Therapiegruppe. Die Fragebögen für das Follow-up wurden in dieser Gruppe zu Beginn der zweitägigen Auffrischungstage 3 Monate nach Therapieende bearbeitet.

Beide Therapieprogramme umfassten Gruppenbehandlungen (medizinisches Schmerzseminar, Trainingstherapie, Entspannung, psychologische Schmerztherapie, Physiotherapieseminar/Edukation, Ergotherapie) und individuelle Behandlungen (medizinische Schmerztherapie, Physio- und Psychotherapie). Die Anzahl der Behandlungsstunden pro Woche (Therapieintensität) war mit etwa 20 h zwischen beiden Gruppen vergleichbar (vgl. *Online-Zusatzmaterial: Tabelle A1*). Das Behandlungsteam bestand aus Ärzt:innen mit der Zusatzbezeichnung spezielle Schmerztherapie, Psycholog:innen/Psychotherapeut:innen, Physiotherapeut:innen, Ergotherapeut:innen und Pflegefachpersonen. Die Behandlung erfolgte in der Regel hauptsächlich gruppenbasiert und nur zu einem geringen Anteil in Einzeltherapiesitzungen. Im Allgemeinen ähnelten sich die Therapie- und Umgebungsbedingungen für beide Therapien hochgradig hinsichtlich therapeutischer Disziplinen, Interventionen und Inhalte.

### Ethik, Ein- und Ausschlusskriterien

Die Studie wurde von der Ethikkommission der Friedrich-Schiller-Universität Jena an der Medizinischen Fakultät positiv votiert (Reg.-Nr.: 2022-2521-Daten). Alle Patient:innen haben der wissenschaftlichen Verwendung der Routinedaten zugestimmt. In die IMST wurden nur volljährige Patient:innen (≥ 18 Jahre), die mindestens 6 Monate unter chronischen Schmerzen litten und multiple erfolglose unimodale Behandlungsversuche durchlaufen haben, eingeschlossen. Die Ausschlusskriterien für die Teilnahme umfassten schwerwiegende psychiatrische Erkrankungen und insuffiziente physische Leistungsfähigkeit.

### Fragebögen und Skalen

Alle Patient:innen bearbeiteten den Deutschen Schmerzfragebogen (DSF, [16]) am ersten (T1, Therapiebeginn) und letzten Tag (T2, Therapieende) der IMST sowie 3 Monate nach Therapieende (T3, 3‑Monats-Follow-up). Im Fokus stand der *Chronic Pain Grade Questionnaire* [13, 25] als Teil des DSF. Hier wurde die durchschnittliche Schmerzintensität der letzten 7 Tage auf einer 11-stufigen numerischen Rating-Skala erfasst (NRS, 0 = kein Schmerz, 10 = stärkster vorstellbarer Schmerz). Die schmerzbedingte Beeinträchtigung hinsichtlich alltäglicher Aktivitäten, Freizeitaktivitäten/Unternehmungen im Familien- oder Freundeskreis und der Arbeitsfähigkeit wurde entsprechend mit 3 Items erfasst (0 = keine Beeinträchtigung, 10 = völlige Beeinträchtigung). Die schmerzbedingte Beeinträchtigungsskala wurde aus dem Mittelwert dieser Items gebildet [13, 25].

### Statistik

#### Primäre und sekundäre Endpunkte

Die primären Endpunkte umfassten Gruppenunterschiede (*KST vs. LTT*) in der schmerzbedingten Beeinträchtigung und durchschnittlichen Schmerzintensität im 3‑Monats-Follow-up (T3).

Als sekundäre Endpunkte wurden die initialen (T1 *vs.* T2) und mittelfristigen (T1 *vs.* T3) Behandlungseffekte (Intragruppenvergleiche) sowie Zwischengruppenvergleiche zu T2 bezüglich der schmerzbedingten Beeinträchtigung und durchschnittlichen Schmerzintensität betrachtet.

#### Matching

Um Vergleichbarkeit in den Basisdaten zu erzielen und Unterschiede in der Stichprobengröße auszuräumen, wurden Patient:innen der *KST*- und *LTT*-Gruppe im Verhältnis 1:1 hinsichtlich Geschlecht, Rückenschmerz, Alter und Schmerzbeeinträchtigung zu Therapiebeginn (T1) gematcht. Die Details können dem *Online-Zusatzmaterial* (Abschnitt B: *Matching*) entnommen werden.

#### Statistische Verfahren

Für dichotome Variablen wurden absolute Häufigkeiten und Prozentwerte bestimmt. Im Falle kontinuierlicher Variablen wurden der Median sowie das erste (Q_1_) und dritte Quartil (Q_3_) ermittelt. Aufgrund des *Matchings* wurden Zwischengruppenvergleiche mittels McNemar-Tests (dichotome Variablen) bzw. Wilcoxon-Vorzeichen-Rang-Tests (kontinuierliche Variablen) angestellt. Dies gilt auch für die Intragruppenvergleiche zwischen den verschiedenen Zeitpunkten. Als Maß der Effektstärke wurde der r‑Koeffizient bestimmt, wobei Werte >0,1 als klein, > 0,3 als moderat und >0,5 als hoch interpretiert werden können [6].

Das Signifikanzniveau wurde auf 5 % gesetzt und zweiseitige *p*-Werte bestimmt. Die Analysen wurden mit R (Version 3.5.1, *R Foundation for Statistical Computing*, Wien, Österreich; [20]) und SPSS (Version 22.0, *IBM Corp.*, Armonk, NY, USA) durchgeführt.

## Ergebnisse

**Abb. 1:**
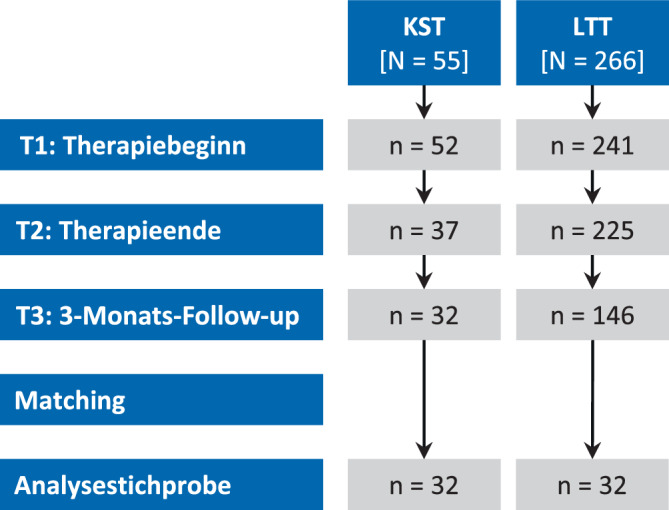
Flussdiagramm der Studie. Dargestellt ist die Anzahl der Patient:innen mit gültigen Fragebogenangaben in der Gruppe mit kurzer stationärer (*KST*) und langer tagesklinischer (*LTT*) Therapie

In der *KST*-Gruppe standen 32 Patient:innen mit vollständigen Angaben für alle Zeitpunkte (T1–T3) zur Verfügung. Von den 146 Patient:innen mit vollständigen Angaben in der *LTT*-Gruppe wurden entsprechend 32 Patient:innen 1:1 gematcht (Abb. 1). Nach dem *Matching* bestanden keine signifikanten Unterschiede hinsichtlich demografischer sowie behandlungs- und schmerzrelevanter Variablen zwischen den beiden Gruppen. Ein hoher Prozentsatz der eingeschlossenen Patient:innen wies auffällige Angst- und Depressionswerte im Screening auf, und mehr als 50 % in beiden Gruppen berichteten über mehr als 5 Jahre anhaltende chronische Schmerzen (Tab. 1 und 2). Die Ergebnisse des *Matchings* (*Zusatzmaterial online: B*) inklusive Vergleichen der gematchten und nichtgematchten *LTT*-Patient:innen (*Online-Zusatzmaterial: C*) sowie der *Drop-out*-Analysen (*Online-Zusatzmaterial: D*) sind online verfügbar.

**Tab. 1:** Demografische und behandlungsassoziierte Variablen zu Behandlungsbeginn

	KST (*N* = 32)	LTT (*N* = 32)	*p*-Wert
Alter (Jahre), Median [IQR]	55,0 [49,2–69,2]	54,5 [50,8–61,8]	0,927^a^
Follow-up-Zeitraum (Tage), Median [IQR]	79,5 [71,0–93,8]	84,5 [73,8–96,0]	0,614^a^
Geschlecht (weiblich), *n* (%)	15 (46,9)	15 (46,9)	0,999^b^
Dauer chronischer Schmerz (≥ 5 Jahre). *n* (%)	19 (59,4)	19 (59,4)	0,999^b^
Chronischer Rückenschmerz (ja), *n* (%)	23 (71,9)	23 (71,9)	0,999^b^
Relevante Angstsymptomatik (>Cut-off)^c^, *n* (%)	14 (43,8)	14 (43,8)	0,999^b^
Relevante Depressionssymptomatik (>Cut-off)^d^, *n* (%)	12 (37,5)	11 (34,4)	0,999^b^

IQR Interquartilsabstand („interquartile range“), KST kurze stationäre Behandlung, LTT lange tagesklinische Behandlung

^a^Wilcoxon-Vorzeichen-Rang-Test

^b^McNemar-Test

^c^KST-Patient:innen: Summenscore der Hospital Anxiety Depression Scale (HADS, [9, 10, 28]): Angst ≥11 oder LTT-Patient:innen: Summenscore der Depression Anxiety and Stress Scale (DASS, [14, 17, 18]): Angst ≥6

^d^KST-Patient:innen: Summenscore der HADS: Depression ≥11 oder LTT-Patient:innen: Summenscore der DASS: Depression ≥10

**Tab. 2:** Zusammenfassende Darstellung der schmerzrelevanten Variablen

		KST (*N* = 32)			LTT (*N* = 32)			Gruppenvergleich	
		Median [IQR]	*p*_T1_	|*r*_T1_|	Median [IQR]	*p*_T1_	|*r*_T1_|	*p*_Gruppe_	|*r*_Gruppe_|
Schmerzbeeinträchtigung (Skala, 0–10)	T1	5,3 [4,0–6,6]	–	–	5,0 [4,0–6,1]	–	–	0,195	0,24
	T2	4,0 [2,2–5,8]	<0,001	0,62	4,0 [2,7–5,4]	<0,001	0,65	0,731	0,06
	T3	6,0 [3,7–7,4]	0,720	0,07	3,8 [2,0–5,2]	0,004	0,51	0,014^a^	0,44
Durchschnittliche Schmerzintensität (NRS, 0–10)	T1	6,0 [5,0–7,0]	–	–	6,0 [4,0–7,0]	–	–	0,950	0,02
	T2	5,0 [4,0–6,0]	0,004	0,59	5,0 [4,0–6,0]	0,001	0,66	0,488	0,13
	T3	6,0 [4,8–7,0]	0,507	0,14	5,0 [3,0–6,0]	0,002	0,60	0,045^a^	0,36

IQR Interquartilsabstand („interquartile range“); KST kurze stationäre Behandlung, LTT lange tagesklinische Behandlung; NRS numerische Rating-Skala; p_Gruppe_ p-Werte der Zwischengruppenvergleiche; p_T1_ p-Werte der Intragruppenvergleiche (T1 vs. T2, T1 vs. T3); r_Gruppe_ Effektstärke der Zwischengruppenvergleiche (> 0,1 kleine, > 0,3 moderate, > 0,5 hohe Effektstärke); r_T1_ Effektstärke der Intragruppenvergleiche; T1 Therapiebeginn; T2 Therapieende; T3 3-Monats-Follow-up

^a^ Bonferroni-Holm-adjustierte p-Werte für die primären Gruppenvergleiche: Schmerzbeeinträchtigung (p = 0,028) und durchschnittliche Schmerzintensität (p = 0,044)

### Primäre Endpunkte und Gruppenvergleiche im Follow-up

**Abb. 2:**
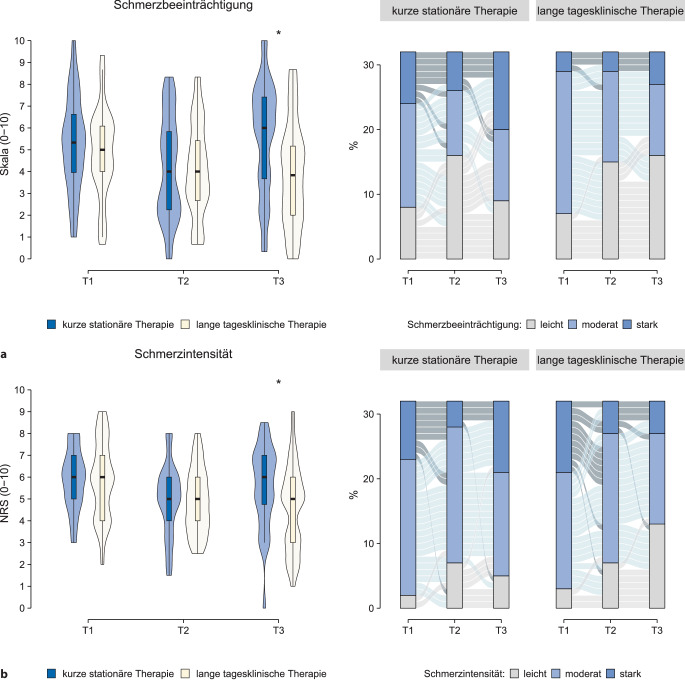
Zusammenfassende Darstellung der (**a**) Schmerzbeeinträchtigung und (**b**) durchschnittlichen Schmerzintensität beider Therapiegruppen für den Therapiebeginn (T1), das Therapieende (T2) und das 3‑Monats-Follow-up (T3). Signifikante Unterschiede (*p* < 0,05; Wilcoxon-Vorzeichen-Rang-Tests) zwischen beiden Gruppen sind mit *Sternchen* markiert. Auf die Darstellung der Intragruppenvergleiche wurde aus Gründen der Übersichtlichkeit verzichtet (siehe Tab. 2). Die *rechten* Teilabbildungen geben die individuellen Verläufe (pro Gruppe *n* = 32 Patient:innen) wieder. Hierfür wurden aus Übersichtlichkeitsgründen die Werte der Schmerzbeeinträchtigung und -intensität jeweils in leicht (0–3), moderat (4–6) und stark (7–10) unterteilt [24]

Im Vergleich zu Patient:innen der *KST* berichteten die Patient:innen der *LTT* über eine signifikant niedrigere Schmerzbeeinträchtigung im 3‑Monats-Follow-up (T3; Tab. 2 und Abb. 2a). Dies galt auch für die durchschnittliche Schmerzintensität (Tab. 2 und Abb. 2b). Die Effektstärken dieser Unterschiede bewegten sich auf moderatem Niveau. Die Unterschiede gingen vor allem auf erneute Verschlechterungen innerhalb der *KST*-Gruppe zwischen T2 und T3 zurück. Während die Patient:innen der *LTT*-Gruppe zu T3 signifikante Unterschiede zu den Ausgangswerten (T1) zeigten (hohe Effektstärken, Tab. 2), konnten für Patient:innen der *KST*-Gruppe keine signifikanten Unterschiede identifiziert werden (Tab. 2).

### Initiale Behandlungseffekte und Gruppenvergleiche

Beide Gruppen wiesen zwischen Therapiebeginn (T1) und -ende (T2) signifikante Behandlungseffekte in der Schmerzbeeinträchtigung und durchschnittlichen Schmerzintensität auf. Die Effektstärken dieser Vergleiche waren moderat bis hoch. Zum Ende der Therapie (T2) bestanden keine signifikanten Unterschiede zwischen den beiden Behandlungsgruppen (Tab. 2).

## Diskussion

In dieser retrospektiven Studie sollte der mittelfristige Erfolg einer kurzen stationären IMST (1 Woche, *KST*) und einer langen tagesklinischen IMST (4 Wochen, *LTT*) bei Patient:innen mit lange bestehenden chronischen Schmerzen und hoher schmerzbedingter Beeinträchtigung am Universitätsklinikum Jena verglichen werden. In beiden Gruppen zeigten sich initial signifikante und mittelgroße Behandlungseffekte (T1 *vs.* T2). Im 3‑Monats-Follow-up konnten diese nur bei Patient:innen der *LTT*-Gruppe nachgewiesen werden.

### Vergleich der Ausgangswerte und kurzfristige Therapieeffekte

Durch das Matching konnte eine hohe Vergleichbarkeit beider Gruppen erzielt werden. Beide Gruppen zeigten nach der Therapie signifikante Verbesserungen. Die Effektstärken sind mit denen anderer interdisziplinärer Behandlungsprogramme mit ähnlicher Dauer und Intensität vergleichbar [19, 23]. Wir konnten keine signifikanten Unterschiede zwischen den beiden Gruppen zu T2 identifizieren, was ähnlich positive, kurzfristige Effekte beider Behandlungssettings nahelegt.

### Mittelfristige Effekte

Patient:innen in der *LTT*-Gruppe zeigten zu T3 eine signifikant geringere schmerzbedingte Beeinträchtigung und Schmerzintensität im Vergleich zu *KST*-Patient:innen. Die Effektstärken dieser Unterschiede lagen im kleinen bis mittleren Bereich. Zu T3 zeigten *LTT*-Patient:innen signifikant bessere Werte im Vergleich zu T1, während sich die Ergebnisse der *KST*-Patient:innen zu T3 wieder auf Ausgangsniveau befanden. Die Effektstärken in der *LTT*-Gruppe decken sich mit denen anderer deutscher IMST-Programme mit vergleichbarer Dauer [19, 23].

Die beiden Therapien unterschieden sich in drei Variablen: (a) der Therapiedosis (Behandlungsdauer) bei vergleichbarer Therapieintensität (Kontaktstunden pro Woche) und vergleichbarer inhaltlicher Ausrichtung der Therapie, (b) der stationären *vs. *tagesklinischen Umgebung und (c) der Gruppengröße.

In Bezug auf die Therapiedosis (Behandlungsdauer) scheint eine Behandlungsdauer von einer Woche (*KST*-Gruppe) für Patient:innen mit initial vergleichbarer Schmerzbeeinträchtigung und Dauer chronischer Schmerzen nicht ausreichend, um eine nicht nur kurzfristige Verbesserung zu gewährleisten. Die konkreten Wirkmechanismen bleiben jedoch offen. Möglich ist ein unmittelbarer Effekt insbesondere durch rasch wirksame Therapieelemente wie Entspannung oder Bewegung, der sowohl bei einer kurzen als auch bei einer langen IMST zum Tragen kommt und zu einer vergleichbaren Verbesserung bei Therapieende führt. Demgegenüber könnten Mechanismen, die potenziell eine längerfristige Stabilisierung ermöglichen, wie eine körperliche Konditionierung und/oder lerntheoretisch begründete Verhaltensänderungen sowie der Transfer funktionaler Schmerzbewältigungsstrategien in den Alltag, eine längere Therapiedauer erfordern. Eine nachträgliche Analyse aller *LTT*-Patient:innen mit vollständigen Angaben für alle Zeitpunkte (T1 bis T3 sowie 6‑ und 12-Monats-Follow-up; *n* = 114) verweist auf einen stabilen Langzeiteffekt des *LTT*-Programms bis zu einem Jahr (*Online-Zusatzmaterial: Abb. E1*). In Anbetracht der vergleichbaren initialen Behandlungseffekte scheint es, dass eine weitere Behandlung das Ausmaß der Verbesserung zwar nicht weiter erhöht, aber notwendig ist, um deren langfristige Stabilität sicherzustellen. Eine Studie aus der Schweiz untersuchte eine einwöchige multidisziplinäre Behandlung in einer ambulanten Einrichtung und konnte auch nach 3 und 12 Monaten signifikante Behandlungseffekte aufzeigen [21]. Jedoch litten diese Patient:innen unter unspezifischen Rückenschmerzen und durchliefen darüber hinaus eine längerfristige ambulante multimodale Schmerztherapie, was eine bedeutende Rolle bei der Aufrechterhaltung der Therapieeffekte gespielt haben könnte [12]. Dies kann als Hinweis gewertet werden, dass bei kurzen IMST-Behandlungen eine ausreichende Nachsorge in Betracht gezogen werden sollte, um die Aufrechterhaltung der Behandlungseffekte zu verbessern.

In Bezug auf die stationäre oder tagesklinische Umgebung (Behandlungssetting) zeigten einige Studien bessere kurzfristige [8, 27] und langfristige [27] Verbesserungen bei stationären im Vergleich zu tagesklinisch oder ambulant durchgeführten Behandlungen. Dies steht im Kontrast zu unseren Befunden. Einerseits könnte eine stationäre Umgebung es Patient:innen ermöglichen, sich vollständig auf die Behandlung zu konzentrieren und den Einfluss alltäglicher Stressfaktoren auf die Behandlungsabläufe zu minimieren. Andererseits könnten tagesklinische oder ambulante Programme es Patient:innen erleichtern, Veränderungen sofort in ihren Alltag zu übertragen, und so eine langfristige Konsolidierung fördern.

Ein weiterer potenzieller Einflussfaktor stellt die Gruppengröße dar, wobei die *KST*-Gruppe kleinere Gruppengrößen aufwies. Kleinere Gruppengrößen können eine höhere Individualisierung der Betreuung ermöglichen und daher die Behandlungseffekte in der *KST*-Gruppe verstärken. Andererseits können sich einige positive Gruppeneffekte möglicherweise nur in größeren Gruppen entfalten.

In Bezug auf die Wirksamkeit der drei oben diskutierten Faktoren (Therapiedosis, stationäre *vs.* tagesklinische Umgebung, Gruppengröße) vermuten wir, dass die Therapiedosis (1 Woche *vs.* 4 Wochen Behandlungsdauer) der stärkste Faktor ist, um die beobachteten mittelfristigen Unterschiede zu erklären.

### Limitationen und Stärken

Die Ergebnisse sind aufgrund des naturalistischen und retrospektiven Designs mit methodischen Einschränkungen verbunden. Die geringe Stichprobengröße, unterschiedliche Behandlungsjahre – *LTT*-Patient:innen wurden später behandelt als *KST*-Patient:innen – und die nichtrandomisierte Zuordnung der Patient:innen reduzieren die interne Validität. Aufgrund der retrospektiven Datenanalyse war der Umfang der analysierten Variablen begrenzt.

Andererseits scheint die externe Validität hoch zu sein, da beide Behandlungsformen unter nichtoptimierten, alltäglichen Bedingungen stattfanden und realitätsnahe Stichproben einer IMST repräsentieren. Eine weitere Stärke besteht in der hohen Vergleichbarkeit der Therapieinhalte, beteiligten Fachdisziplinen und Charakteristika der Patient:innen zu Beginn der Therapie.

### Schlussfolgerung

Unsere Ergebnisse deuten darauf hin, dass initiale Behandlungseffekte auch nach einer kurzen IMST (niedrige Therapiedosis) auftreten. Um eine langfristige Stabilität der Behandlungseffekte zu gewährleisten, scheint aber bei stark ausgeprägten chronischen Schmerzen eine längere Behandlungsdauer (höhere Therapiedosis) vorteilhaft zu sein. Die methodischen Einschränkungen der aktuellen Studie lassen jedoch keine Verallgemeinerung dieser Interpretation zu. Die Studie verdeutlicht, welche methodischen Schwierigkeiten bei der Untersuchung unterschiedlicher Dosierungsaspekte und Behandlungssettings berücksichtigt werden sollten.

## Fazit für die Praxis

- Die IMST zeigt moderate bis hohe Wirksamkeitseffekte bereits frühzeitig in der Behandlung.
- Zur Gewährleistung von Stabilität der Behandlungseffekte scheint bei vergleichbarer Dauer der Schmerzsymptomatik und Höhe der schmerzbedingten Beeinträchtigung eine höhere Therapiedosis (längere Behandlungsdauer) vorteilhaft zu sein.
- Zur schlussendlichen wissenschaftlichen Klärung dieser Befunde sind randomisiert-kontrollierte Studien notwendig, bei denen Patient:innen mit unterschiedlichen Chronifizierungsgraden verschiedenen Therapiedosen (Behandlungsdauer) bei möglichst konstanten Behandlungssettings und Therapieinhalten zugewiesen werden.

## Supplementary Information

A: Vergleich der Behandlungen und Behandlungsstunden (Tab. A1); B: *Matching* (Tab. B1 und B2); C: Vergleich der gematchten und ungematchten LTT-Gruppen (Tab. C1 und C2); D: Drop-out-Analyse (Tab. D1); E: Langzeiteffekte der LTT-Behandlung (Abb. E1)

## Data Availability

Die Daten, die die Ergebnisse dieser Publikation stützen, sind auf begründete Anfrage bei den korrespondierenden Autoren erhältlich.
