# Supplementary material for: Interdisciplinary multimodal pain therapy: does the dose make a difference? A comparison from routine clinical care
Source: Schmerz. 2024 Oct 9;40(2):105–12. [Article in German] doi: 10.1007/s00482-024-00838-6 (PMC13004731; doi:10.1007/s00482-024-00838-6)
Supplement: Supplementary file 1 — A: Vergleich der Behandlungen und Behandlungsstunden (Tab. A1); B: Matching (Tab. B1 und B2); C: Vergleich der gematchten und ungematchten LTT-Gruppen (Tab. C1 und C2); D: Drop-out-Analyse (Tab. D1); E: Langzeiteffekte der LTT-Behandlung (Abb. E1) [file 482_2024_838_MOESM1_ESM.pdf]

Online Zusatzmaterial

A Vergleich der Behandlungen und Behandlungsstunden

Eine IMST wurde in einem stationären Setting an 7 Behandlungstagen (1 Woche) durchgeführt und wird als KST (kurz stationär) bezeichnet. Sie fand zwischen Januar 2009 und November 2010 statt. Die andere IMST wurde in einem Tagesklinik-Setting an 20 Behandlungstagen (4 Wochen) durchgeführt und wird als LTT (lang tagesklinisch) bezeichnet. Die LTT-IMST wurde zwischen Juni 2013 und März 2017 durchgeführt. In Bezug auf die Behandlungsstunden während einer Woche Therapie sind beide Settings gleich (siehe Tabelle A1).

Tab. A1 Behandlungsstunden für verschiedene Behandlungen während einer Behandlungswoche in der KST- und der LTT-Gruppe

		**Kontaktstunden**	
		**KST**	**LTT**
Gruppenbehandlungen	**Medizinisches Schmerzseminar**	1,5	1,5
	**Trainingstherapie**	5	5
	**Entspannung**	1,5	3
	**Psychologische Schmerztherapie**	3	4
	**Physiotherapieseminar - Edukation**	1	1
	**Ergotherapie**	-	1
Individuelle Behandlungen	**Physiotherapie**	5	1
	**Psychotherapie**	1	1
	**Medizinische Schmerztherapie**	1,5	1,5
**SUMME**		**19,5**	**19**

B *Matching*

Um Vergleichbarkeit in den Basisdaten zu erreichen und Unterschiede in der Stichprobengröße zu reduzieren, wurden KST- und LTT-Patient:innen im Verhältnis 1:1 gematcht. Der Deutsche Schmerzfragebogen (DSF) wurde im Jahr 2011 überarbeitet, und die Hospital *Anxiety and Depression Scale* (HADS) [1, 2] wurde durch die *Depression Anxiety and Stress* Skala (DASS) [3, 4] ersetzt. Um einheitliche Skalen für die Basiskennzeichnung innerhalb der KST-Gruppe (HADS) und LTT-Gruppe (DASS) zu gewährleisten, wurden LTT-Patient:innen, die die HADS abgeschlossen hatten (n = 28), vom *Matching*-Verfahren ausgeschlossen.

Zunächst wurde nach Geschlecht und Vorhandensein von chronischen Rückenschmerzen (ja *vs.* nein) gematcht. Anschließend wurden die Patient:innen gemäß Alter und der Schmerz-bedingten

Beeinträchtigung zum Zeitpunkt T1 innerhalb der jeweiligen Untergruppen (weiblich und Rückenschmerzen: nein | weiblich und Rückenschmerzen: ja | männlich und Rückenschmerzen: nein | männlich und Rückenschmerzen: ja) durch *nearest neighbor Matching* abgeglichen. Das *Matching* wurde mit dem „matchIt"-Paket in R (Version 3.5.1, *R Foundation for Statistical Computing*, Wien, Österreich) durchgeführt.

Tab. B1 Baseline-Analysen (Therapiebeginn) der vollständigen LTT- und KST-Gruppe der IMST

		**KST (n=32)**			**LTT: alle (n=118)**			
		**Median**	**Q_1**	**Q_3**	**Median**	**Q_1**	**Q_3**	***p^1***
**Alter**	(Jahre)	55,0	47,8	69,8	52,0	45,0	58,3	,066
**Schmerzbedingte Beeinträchtigungsskala**	(NRS)	5,3	3,9	6,9	5,3	4,0	6,7	,978
**Durchschnittliche Schmerzintensität**	(NRS)	6,0	5,0	7,0	6,0	4,8	7,0	,671
**Maximale Schmerzintensität**	(NRS)	7,5	7,0	9,0	8,0	7,0	8,0	,324
**Follow-up Zeitraum**	(Tage)	79,5	71,0	95,3	94,0	71,8	99,0	,216
		**n**	**%**		**n**	**%**		***p^2***
**Geschlecht**	(weiblich)	15	46,9		89	75,4		**,002**
**Angst**	(ja[3])	14	43,8		44	37,3		,506
**Depression**	(ja[4])	12	37,5		34	28,8		,345
**Dauer chronischer Schmerz**	(≥ 5 Jahre)	19	59,4		60	50,8		,391
**Chronischer Rückenschmerz**	(ja)	23	71,9		51	43,2		**,004**

Demografische, behandlungsbezogene und schmerzbezogene Patient:innendaten beider Gruppen. Kontinuierliche Daten werden als Median, erstes und drittes Quartil (Q_1, Q_3) angegeben. Dichotome Daten werden als Anzahl (n) und Prozent (%) angegeben. Die p-Werte der Gruppenvergleiche werden in der letzten Spalte (*p*) angezeigt. Fettgedruckte *p*-Werte verweisen auf signifikante Gruppenunterschiede.

[1] *p*-Werte der Mann-Whitney-U-Test; [2] *p*-Werte der Chi-Quadrat-Tests; [3] Summenscore der *Hospital Anxiety Depression Scale* (HADS): Angst >10 oder Summenscore der *Depression, Anxiety and Stress Scale* (DASS): Angst >6; Summenscore HADS: Depression >10 oder Summenscore der DASS: Depression >10; **KST** kurze stationäre Therapie; **LTT** lange tagesklinische Behandlung; **NRS** numerische Rating Skala (0–10)

Tab B2 Ergebnisse des Matchings

	KST		LTT: alle			LTT: gematcht			
**"exaktes" *Matching***									
	**KST (n=32)**		**LTT: alle (n=118)**			**LTT: gematcht (n=32)**			
	**n**	**%**	**n**	**%**	**Δ%**	**n**	**%**	**Δ%**	**% Verbesserung**
**Geschlecht** (weiblich)	15	46,9	89	75,4	28,5	15	46,9	0,0	**100,0**
**Rückenschmerz** (ja)	23	71,9	51	43,2	28,7	23	71,9	0,0	**100,0**
***nearest neighbor Matching* innerhalb der Subgruppen**									
	**KST**		**LTT: alle**			**LTT: gematcht**			
**weiblich & Rückenschmerz: ja**	**MW**		**MW**	**SD**	**ΔMW**	**MW**	**SD**	**ΔMW**	**% Verbesserung**
**Alter (Jahre)**	59,8		54,0	10,2	5,9	59,3	11,1	0,5	
**Schmerzbedingte Beeinträchtigungsskala (0–10)**	5,2		5,2	1,7	0,0	5,0	1,4	0,3	
**Distanz:**	**0,3**		**0,2**	**0,1**	**0,1**	**0,3**	**0,1**	**0,0**	**82,9**
**weiblich & Rückenschmerz: nein**	**MW**		**MW**	**SD**	**ΔMW**	**MW**	**SD**	**ΔMW**	**% Verbesserung**
**Alter (Jahre)**	62,0		51,4	10,8	10,6	60,7	12,6	1,3	
**Schmerzbedingte Beeinträchtigungsskala (0–10)**	6,2		5,1	2,0	1,1	5,6	4,0	0,7	
**Distanz:**	**0,2**		**0,0**	**0,0**	**0,2**	**0,1**	**0,1**	**0,1**	**56,5**
**männlich & Rückenschmerz: ja**	**MW**		**MW**	**SD**	**ΔMW**	**MW**	**SD**	**ΔMW**	**% Verbesserung**
**Alter (Jahre)**	56,8		50,3	9,9	6,6	54,9	8,1	1,9	
**Schmerzbedingte Beeinträchtigungsskala (0–10)**	5,5		5,0	2,2	0,4	4,5	2,4	1,0	
**Distanz:**	**0,5**		**0,4**	**0,1**	**0,1**	**0,4**	**0,1**	**0,1**	**40,5**
**männlich & Rückenschmerz: nein**	**MW**		**MW**	**SD**	**ΔMW**	**MW**	**SD**	**ΔMW**	**% Verbesserung**
**Alter (Jahre)**	49,7		46,2	12,3	3,4	50,7	7,9	-1,0	
**Schmerzbedingte Beeinträchtigungsskala (0–10)**	4,6		6,0	2,2	-1,4	5,0	2,0	-0,4	
**Distanz:**	**0,4**		**0,3**	**0,1**	**0,1**	**0,4**	**0,1**	**0,0**	**73,3**

Ergebnisse des sequenziellen *Matching*-Verfahrens für Patient:innen in der KST- im Vergleich zur LTT-Gruppe. Zunächst wurden die Patient:innen hinsichtlich des Geschlechts und des Vorhandenseins chronischer Rückenschmerzen exakt abgeglichen. Anschließend wurden die Patient:innen innerhalb der Untergruppen (weiblich und keine Rückenschmerzen | weiblich und Rückenschmerzen | männlich und keine Rückenschmerzen | männlich und Rückenschmerzen) anhand des Alters und der schmerzbedingten Beeinträchtigungsskala mittels *nearest neighbor Matching* abgeglichen.

KST kurze stationäre Therapie; **LTT** lange tagesklinische Behandlung; **MW** Mittelwert, **SD** Standardabweichung

C Vergleich der gematchten und ungematchten LTT-Gruppen

Tab. C Vergleich der kontinuierlichen Variablen zwischen gematchten und ungematchten Patient:innen der langen tagesklinischen Therapiegruppe (LTT)

		LTT: gematcht (n=32)			LTT: nicht gematcht (n=86)			
**T1: Therapiebeginn**		**Median**	**Q_1**	**Q_3**	**Median**	**Q_1**	**Q_3**	***p*-Wert**
**Schmerbedingte Beeinträchtigungsskala**	(0-10)	5,0	4,0	6,3	5,3	4,3	7,0	,177
**Durchschnittliche Schmerzintensität**	(0-10)	6,0	4,0	7,0	6,0	5,0	7,0	,700
**Maximale Schmerzintensität**	(0-10)	7,5	7,0	8,0	8,0	7,0	8,0	,823
**Angst**	(0-21)	3,5	2,0	8,8	4,0	1,0	7,0	,588
**Depression**	(0-21)	6,5	3,3	12,8	7,0	4,0	10,0	,879
**T2: Therapieende**		**Median**	**Q_1**	**Q_3**	**Median**	**Q_1**	**Q_3**	***p*-Wert**
**Schmerbedingte Beeinträchtigungsskala**	(0-10)	4,0	2, 7	5,6	4,5	2,7	6,1	,509
**Durchschnittliche Schmerzintensität**	(0-10)	5,0	4,0	6,0	5,0	4,0	6,0	,614
**Maximale Schmerzintensität**	(0-10)	7,0	6,3	8,0	7,0	6,0	8,0	,389
**Angst**	(0-21)	3,0	1,0	6,8	2,5	1,0	5,0	,266
**Depression**	(0-21)	4,0	0,0	8,0	3,0	1,0	6,0	,857
**T3: 3 Monate Follow-up**		**Median**	**Q_1**	**Q_3**	**Median**	**Q_1**	**Q_3**	***p*-Wert**
**Schmerbedingte Beeinträchtigungsskala**	(0-10)	3,8	2,0	5,5	4,5	2,7	6,1	,526
**Durchschnittliche Schmerzintensität**	(0-10)	5,0	3,0	6,0	5,0	4,0	6,0	,556
**Maximale Schmerzintensität**	(0-10)	7,0	5,0	8,0	7,0	6,0	8,0	,593
**Angst**	(0-21)	3,0	1,0	7,8	3,0	1,0	6,0	,208
**Depression**	(0-21)	5,0	1,3	8,8	6,0	1,8	8,0	,939

Median sowie erstes und drittes Quartil (Q_1/Q_3) sowie *p*-Werte der Gruppenvergleiche unter Verwendung von Mann-Whitney-U-Tests sind in der letzten Spalte dargestellt.

Tab. C1 Vergleich der kategorialen Variablen zwischen gematchten und ungematchten Patient:innen der langen tagesklinischen Therapiegruppe (LTT).

			LTT: gematcht (n=32)		LTT: ungematcht (n=86)		
			**n**	**%**	**n**	**%**	***p*-Wert**
**Angst**	(ja[1])	T1	14	43,8	30	34,9	,376
		T2	12	37,5	18	20,9	,066
		T3	13	40,6	23	26,7	,145
**Depression**	(ja[2])	T1	11	34,4	23	26,7	,416
		T2	6	18,8	8	9,3	,158
		T3	7	21,9	15	17,4	,583

Anzahl (n) und Prozent (%) der Patient:innen mit klinisch relevanter Angst- oder Depressionssymptomatik (**T1** Therapiebeginn; **T2** Therapieende; **T3** 3 Monate Follow-up). Gruppenunterschiede wurden mit Chi-Quadrat-Tests analysiert.

[1] Summenscore der *Depression, Anxiety and Stress Scale* (DASS): Angst >6
[2] Summenscore der *Depression, Anxiety and Stress Scale* (DASS): Depression >10

D *Drop-out*-Analyse

Tab. D1 Ergebnisse der Drop-out Analyse für kontinuierliche Variablen.

		Dropout T2–T3: ja (n=62)			Dropout T2–T3: nein (n=150)			
**T1: Therapiebeginn**		**Median**	**Q_1**	**Q_3**	**Median**	**Q_1**	**Q_3**	***p*-Wert**
**Schmerzbedingte Beeinträchtigungsskala**	(0-10)	4,7	2,9	7,0	5,3	4,0	6,7	,118
**Durchschnittliche Schmerzintensität**	(0-10)	5,5	4,0	7,0	6,0	5,0	7,0	,412
**Maximale Schmerzintensität**	(0-10)	7,0	6,0	8,0	8,0	7,0	8,1	,357
**T2: Therapieende**		**Median**	**Q_1**	**Q_3**	**Median**	**Q_1**	**Q_3**	***p*-Wert**
**Schmerzbedingte Beeinträchtigungsskala**	(0-10)	3,2	2,0	5,8	4,3	2,7	6,0	,218
**Durchschnittliche Schmerzintensität**	(0-10)	5,0	3,9	6,1	5,0	4,0	6,0	,649
**Maximale Schmerzintensität**	(0-10)	7,0	6,0	8,0	7,0	6,0	8,0	,763
**T1 *vs.* T2**		**Median**	**Q_1**	**Q_3**	**Median**	**Q_1**	**Q_3**	***p*-Wert**
**Schmerzbedingte Beeinträchtigungsskala**	(0-10)	-0,7	-1,7	0,3	-0,9	-2,0	0,0	,476
**Durchschnittliche Schmerzintensität**	(0-10)	-1,0	-2,0	0,5	-0,5	-2,0	0,0	,812
**Maximale Schmerzintensität**	(0-10)	0,0	-1,1	0,1	0,0	-1,1	0,1	,593

Median sowie erstes und drittes Quartil (Q_1/Q_3) der Patient:innen mit oder ohne Nachuntersuchung (T3) für beide Therapiegruppen. Die *p*-Werte (*p*) der Gruppenvergleiche unter Verwendung von Mann-Whitney-U-Tests sind in der letzten Spalte dargestellt.

Tab. D2 Ergebnisse der Drop-out Analyse für kategoriale Variablen.

			Dropout T2–T3: ja (n=62)		Dropout T2–T3: nein (n=150)		
			**n**	**%**	**n**	**%**	***p*-Wert**
**Angst**	(ja[1])	T1	25	40,3	58	38,7	,822
		T2	12	19,4	38	25,5	,339
**Depression**	(ja[2])	T1	21	33,9	46	30,7	,648
		T2	11	17,7	22	14,8	,588

Anzahl (n) und Prozent (%) der Patient:innen mit klinisch relevanter Angst- oder Depressionssymptomatik (**T1** Therapiebeginn; **T2** Therapieende). Gruppenunterschiede wurden mit Chi-Quadrat-Tests analysiert.

[1] Summenscore der *Hospital Anxiety Depression Scale* (HADS): Angst >10 oder Summenscore der *Depression, Anxiety and Stress Scale* (DASS): Angst >6; [2] Summenscore der HADS: Depression >10 oder Summenscore DASS: Depression >10

E Langzeit-Effekte der LTT-Behandlung

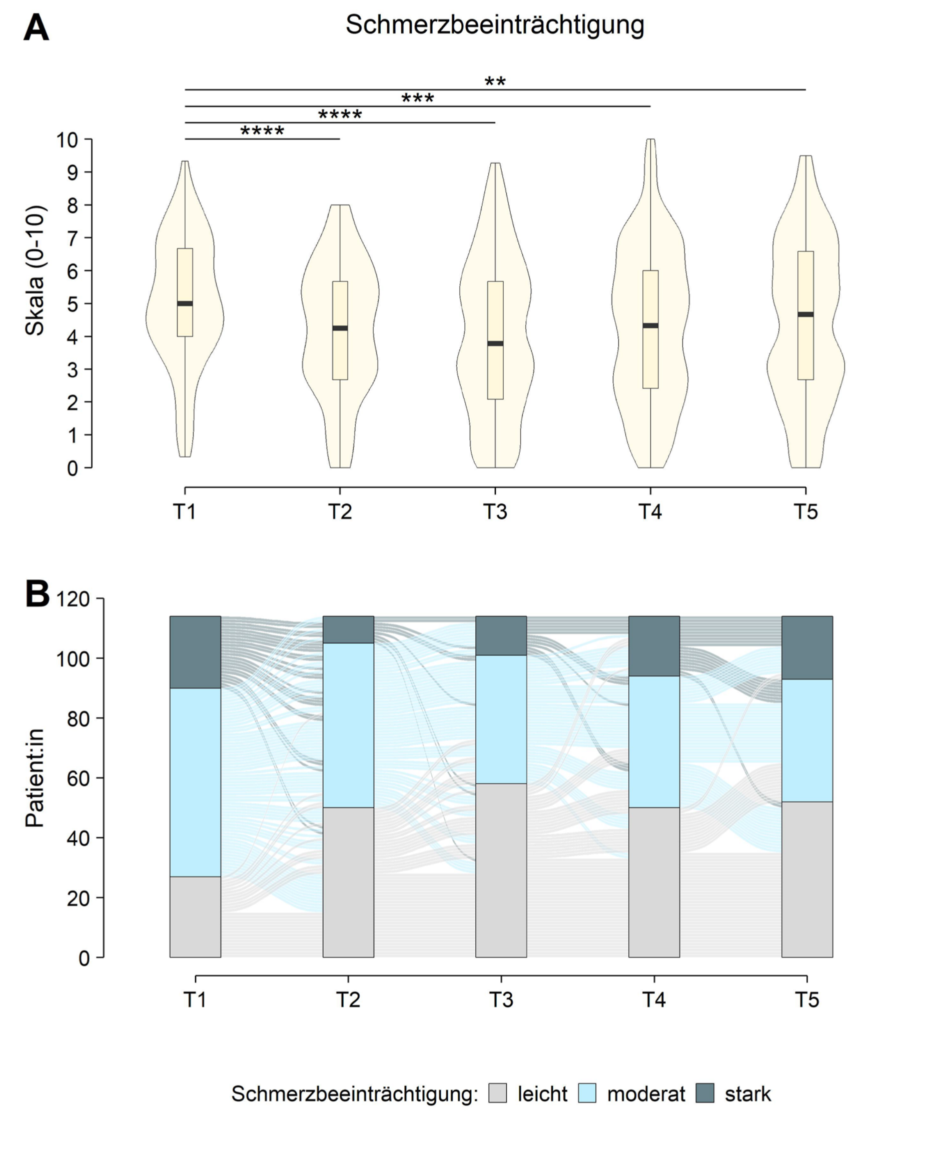

Abb. E1 (A) Zusammenfassende Darstellung der Schmerzbeeinträchtigung für die 114 Patient:innen in der langen tagesklinischen Behandlungsgruppe (LTT) mit vollständigen Datensätzen für T1 (Therapiebeginn), T2 (Therapieende), 3 Monate (T3, Median=3, $Q_{1–3}$: 2–3 Monate), 6 Monate (T4, Median=6, $Q_{1–3}$: 6–7 Monate) und 12 Monate Follow-up (T5, Median=12, $Q_{1–3}$: 12–12 Monate). Signifikante Unterschiede zu den T1-Daten in den Wilcoxon-Vorzeichen-Rang-Tests sind mit Sternen markiert (Bonferroni-Holm-korrigierte p-Werte: ** p<0,01; *** p<0,001; **** p<0,0001; Effektstärken T1–T2: r=0,52; T1–T3: r=0,50; T1–T4: r=0,38; T1–T5: r=0,29). (B) Individuelle Verläufe (n=114) der Schmerzbeeinträchtigung. Hierfür wurden aus Übersichtlichkeitsgründen die Werte jeweils in leicht (0–3), moderat (4–6) und stark (7–10) unterteilt [5].

Literaturverzeichnis

1. Herrmann-Lingen C, Buss U, Snaith RP (1995) Hospital Anxiety and Depression Scale - deutsche Version (HADS-D). Verlag Hans Huber, Bern

2. Hinz A, Brahler E (2011) Normative values for the Hospital Anxiety and Depression Scale (HADS) in the general German population. J Psychosom Res 71:74-78

3. Lovibond PF, Lovibond SH (1995) The Structure of Negative Emotional States - Comparison of the Depression Anxiety Stress Scales (Dass) with the Beck Depression and Anxiety Inventories. Behav Res Ther 33:335-343

4. Nilges P, Essau C (2015) Depression, anxiety and stress scales. DASS-A screening procedure not only for pain patients. Schmerz 29:649-657

5. Treede R-D, Rief W, Barke A et al. (2019) Chronic pain as a symptom or a disease: the IASP Classification of Chronic Pain for the International Classification of Diseases (ICD-11). PAIN 160:19-27
